# Supplementary material for: Complexome profiling of the Chlamydomonas psb28 mutant reveals TEF5 as an early PSII assembly factor
Source: Plant Cell. 2025 Mar 17;37(6):koaf055. doi: 10.1093/plcell/koaf055 (PMC12164590; doi:10.1093/plcell/koaf055)
Supplement: koaf055_Supplementary_Data [file koaf055_supplementary_data.zip › psb28_interactive.zip!/plots/intensity/qualityPlots/peptides/FKB19_Cre12.g534700.t1.2.html]

### Cre12.g534700.t1.2

Trivial name: FKB19  
SeqCoverage [median]: 43.30  
Mass: 23.46 kDa  
  
 

| Sequence | MissedCleavage | Mass [Da] | wtIntensity | muIntensity | Score |
| --- | --- | --- | --- | --- | --- |
| ALDFVLANR | 0 | 1017.56 | 663.54 | 7352.36 | 144.38 |
| DYVTTPSGLQYQDIK | 0 | 1726.84 | 0.00 | 418.08 | 93.26 |
| FVLGEGK | 0 | 748.41 | 0.00 | 995.32 | 70.91 |
| GSSFNDDNKDFYR | 1 | 1563.66 | 534.56 | 4141.52 | 62.34 |
| IIVPVELGYPEDNWRK | 1 | 1927.02 | 0.00 | 2098.19 | 31.39 |
| LGPKPSTFAGDR | 1 | 1244.65 | 1012.01 | 5004.73 | 88.80 |
| PSTFAGDR | 0 | 849.40 | 0.00 | 278.66 | 86.10 |
| VIPAFEEAVADMKPGGIR | 1 | 1898.99 | 0.00 | 3870.99 | 62.86 |
| VIPAFEEAVADMKPGGIRR | 2 | 2055.09 | 0.00 | 1751.09 | 43.02 |
| VKDYVTTPSGLQYQDIK | 1 | 1954.00 | 0.00 | 3285.72 | 79.97 |
|  |  |  | 2210.1 | 29196.7 |  |
